# A systematic review and meta-analysis of selected motor learning principles in physiotherapy and medical education

**DOI:** 10.1186/s12909-016-0538-z

**Published:** 2016-01-15

**Authors:** Martin Sattelmayer, Simone Elsig, Roger Hilfiker, Gillian Baer

**Affiliations:** Queen Margaret University, School of Health Sciences, Physiotherapy, Edinburgh, Scotland; University of Applied Sciences and Arts Western Switzerland Valais (HES-SO Valais-Wallis), School of Health Sciences, Leukerbad, Switzerland

**Keywords:** Procedural skills, Clinical skills, Motor learning, Practice schedule, Mental practice, Feedback

## Abstract

**Background:**

Learning of procedural skills is an essential component in the education of future health professionals. There is little evidence on how procedural skills are best learnt and practiced in education. There is a need for educators to know what specific interventions could be used to increase learning of these skills. However, there is growing evidence from rehabilitation science, sport science and psychology that learning can be promoted with the application of motor learning principles. The aim of this review was to systematically evaluate the evidence for selected motor learning principles in physiotherapy and medical education. The selected principles were: whole or part practice, random or blocked practice, mental or no additional mental practice and terminal or concurrent feedback.

**Methods:**

CINAHL, Cochrane Central, Embase, Eric and Medline were systematically searched for eligible studies using pre-defined keywords. Included studies were evaluated on their risk of bias with the Cochrane Collaboration’s risk of bias tool.

**Results:**

The search resulted in 740 records, following screening for relevance 15 randomised controlled trials including 695 participants were included in this systematic review. Most procedural skills in this review related to surgical procedures. Mental practice significantly improved performance on a post-acquisition test (SMD: 0.43, 95 % CI 0.01 to 0.85). Terminal feedback significantly improved learning on a transfer test (SMD: 0.94, 95 % CI 0.18 to 1.70). There were indications that whole practice had some advantages over part practice and random practice was superior to blocked practice on post-acquisition tests. All studies were evaluated as having a high risk of bias. Next to a possible performance bias in all included studies the method of sequence generation was often poorly reported.

**Conclusions:**

There is some evidence to recommend the use of mental practice for procedural learning in medical education. There is limited evidence to conclude that terminal feedback is more effective than concurrent feedback on a transfer test. For the remaining parameters that were reviewed there was insufficient evidence to make definitive recommendations.

**Electronic supplementary material:**

The online version of this article (doi:10.1186/s12909-016-0538-z) contains supplementary material, which is available to authorized users.

## Background

Learning of procedural skills is an essential component in in the education of future medical and physiotherapy professionals [1]. Teaching of procedural skills is traditionally based in the field of surgical education, but has moved in the last decades to almost every discipline in medicine and health professions education (HPE) [2]. Procedural skills are taught in many healthcare areas, for example in nursing education (e.g. intubation) and physiotherapy education (e.g. joint mobilisation). Procedural skills in HPE are highly context specific and learners need to adapt to various conditions [3]. Procedural skills in the context of HPE are often classified under the umbrella term “clinical skills” [4]. However, some authors refer to “psychomotor tasks” [5] where others also include tasks such as communication skills and treatment skills under “procedural skills”.

Training in HPE is expensive and therefore training should be effective [6]. To improve effectiveness, educators need to know what specific educational interventions could be used to enhance learning of these procedural skills. For this review, we defined procedural skills as “a motor skill involving a series of discrete responses each of which must be performed at the appropriate time in the appropriate sequence” [7]. A procedure can serve different purposes (e.g. it may be a diagnostic or therapeutic procedure). Procedures can be simple tasks with only a few parts or they can involve complex sequences of multiple activities that are linked together. Each procedure requires acquisition of unique motor skills. Because of this similarity we are using the terms procedural skills and motor skills interchangeably in this review. We appraised learning of procedures from the study of motor learning, which is the study of the acquisition of motor skills or the performance improvement of learned or highly practiced motor skills [8].

Learning is defined as: “A change in the capability of a person to perform a skill that must be inferred from a relatively permanent improvement in performance as a result of practice”[8, p. 257]. However, this changed capability in motor learning is not directly measurable, because the changes responsible for motor learning are complex processes within the central nervous system. Therefore, change can be inferred by sustained improved performance, but measurement with standardised educational tests is difficult.

Brydges and colleagues [9] argue that programmes in HPE concentrate efforts to improve aspects of education such as evaluation methods. In contrast, very little consideration is given to and there is little evidence on how procedural skills are best taught and practiced in education. There is however, growing evidence from rehabilitation science, sport science and psychology that motor learning can be promoted with the application of motor learning principles (e.g. [10–12]). Wulf et al. [13] proposed that motor-learning principles should be applied to the field of HPE. They argue that procedural skills are an essential component in many curricula. Furthermore, major changes on how procedures are learned have recently been proposed (among others a shift away from traditional approaches of procedural skill learning in HPE such as the Halstedian “see one, do one, teach one” training and involvement of new technologies during procedural learning), and recent evidence questions some traditional assumptions regarding skills learning (e.g. the effectiveness of different practice schedules) [13]. In addition, the way instructions and feedback are given is noted to be not in accordance with research evidence [12]. This emerging interest in how procedural skills are taught formed the basis for this review.

In considering the learning of procedural skills, there are a number of clearly defined parameters within the sports science literature. These mostly look at how to structure practice; how and when to provide feedback and how and when to integrate mental practice alongside physical practice [8]. In undertaking this systematic review, the authors reviewed the literature in relation to motor skill acquisition principles that had some published evidence and that were deemed relevant to HPE. Motor learning texts [8, 14] were searched for eligible principles to include. Firstly, selection of principles was based on available evidence in HPE (i.e. at least one published RCT). Secondly, it should be possible to apply the principle without considerable technical equipment. Within this review four motor learning principles were deemed relevant:

- Part practice or whole practice
- Random practice or blocked practice
- Mental practice
- Augmented feedback (terminal feedback or concurrent feedback)

For clarity, a brief definition of each principle is provided below and a practical application of the principles is presented as an Additional file 1.

### Part practice or whole practice

A procedural skill can be trained with different practice schedules. Learning a procedure in a part practice condition requires breaking this procedure into several fundamental movement segments. After mastering the isolated parts the learner proceeds to practice the parts together. In whole practice the entire procedure is taught in a serial order and as a whole entity [9].

### Random practice or blocked practice

In random practice, multiple components of a procedural skill are practiced in a single session in a random order. Conversely, blocked practice, requires skills to be practiced in closed blocks and progression to the next skill in the block occurs after a predefined amount of practice. Organisation of the practice schedule into random practice may increase the level of difficulty during skill learning and can therefore have negative effects on the performance of the procedure on post-acquisition tests (i.e. a test immediately after an intervention) but may increase performance on retention and transfer tests [15]. It was hypothesised that the increased performance may be caused by more intensive motor planning operations during random practice conditions, which can lead to better memory retrieval on retention and transfer tests [16].

### Mental practice

Mental practice is a method for learning a procedure without actually physically performing it. Mental practice relates to mental rehearsal in this review. This doesn’t cover other practice conditions such as relaxation or meditation exercises. Mental practice may involve exercises such as thinking about the procedure and its parts but mental practice may also include different imagery techniques (with the purpose to maximise equivalence with physical practice, e.g. instruction mode or position of the learner) [17].

### Augmented Feedback (terminal feedback or concurrent feedback)

Augmented feedback is defined as “information about a performance that supplements sensory feedback and comes from a source external to the performer” ([8], p. 344). In educational settings the external source might be an educator. But augmented feedback can also be generated with a computer. An important question in HPE with controversial opinions is the timing of the augmented feedback [8]. When concurrent feedback is used the learner receives augmented feedback during the movement. In contrast terminal feedback is provided after the procedure is completed.

### Learning versus performance

Several possible methods exist to evaluate the performance of a learner. Firstly, “post-acquisition tests” measure performance immediately at the end of an intervention designed to improve learning. This method is valid to measure a change in performance, but because of the immediacy of testing, caution is required in interpreting whether learning has occurred as the resultant performance reflects a potentially temporary situation and should not be associated with a relatively permanent change associated with learning. Rather than testing learning immediately after the teaching and practicing of a new skill, researchers advocate undertaking a “retention test” during which time a rest period (usually a few hours or days) is inserted between the last practice trial and the retention test. The idea of this resting period is that non-permanent effects of the intervention are eliminated and only the permanent changes, which might be indicative of learning are measured. Lastly, researcher may use a “transfer test”. During transfer tests, the ability of the learner to adapt the newly learnt procedural skill to a different situation is tested (e.g. a similar task is practiced in a novel situation under time constraints), often at a time-point distant to the skill acquisition phase. The assumption behind transfer tests is that the adaptability of a learner to a variety of circumstances increases with the degree of learning [8]. This implies that in the situation when learning has not occurred, but there has been a temporary improvement in performance on a post-acquisition test an individual may be unable or will only have limited ability to adapt a procedure to a new situation. In contrast a skilled person, who has acquired genuine learning will be able to adapt the procedural skill to new demands.

### Aim

The aim of this review was to evaluate the evidence for the effectiveness of using motor learning principles to promote learning of procedural skills in physiotherapy and medical education.

## Methods

### Selection of studies

The following criteria were used to include or exclude studies:

### Inclusion criteria

#### Population

We were interested in studies that included students in medical and physiotherapy education. This included undergraduate and postgraduate students.

### Intervention

The intervention had to use at least one of the four motor learning principles identified above with the aim of improving the learning of procedural skills.

### Outcomes

The primary outcome of this review was learning of a procedural skill measured by performance of the procedure. Two different kinds of performance tests for measuring outcome were deemed eligible for this review.

1. The first were procedural specific checklists and the second were global rating scales. Procedural specific checklists identify important parts of a procedure and every task is usually scored on a dichotomous scale. Global rating scales are designed with a range of response options and can be used for more than one procedure. Both types of measurement instruments are frequently used in education research and are valid outcome measures to evaluate the performance of a procedure [18]. Norcini [19] reported a strong correlation between both types of measurements.
2. The second outcome of this systematic review was movement duration. Especially, in surgery movement duration is an important measure for procedure performance [20]. Only studies with at least one of these outcomes were included.

Outcomes taken either during post-acquisition, retention or transfer tests were considered appropriate for this review.

### Design

Randomised controlled studies were included.

### Search methods for identification of studies

The following electronic databases were systematically searched for eligible studies: CINAHL, Cochrane CENTRAL, EMBASE, ERIC and Medline. There was no limit on recency of publication and language of publication. The search string is presented in Table 1. All retrieved papers were imported in an electronic literature management system. In a first step duplicates were removed. In a second step one author (MS) screened titles and abstracts of the remaining records and excluded all irrelevant papers. Lastly, all remaining records were read as full-text articles by two reviewers (SE and MS) and included into the analysis if appropriate. Furthermore, the reference lists of the included articles were hand-checked for additional relevant articles. Two reviewers (SE and MS) independently performed the data extraction. Disagreements between the reviewers (SE and MS) were solved by discussion.

**Table 1:** Search strategy

Population	Intervention	Outcome
medical education OR education, medical [Mesh] OR physiotherapy education OR physical therapy education OR health professions education OR healthcare education	whole practice OR part practice OR random practice OR blocked practice OR whole task OR part task OR random task OR blocked task OR practice schedule OR practice distribution OR mental imagery OR mental practice OR mental rehearsal OR augmented feedback OR knowledge of results OR knowledge of performance OR terminal feedback OR concurrent feedback OR focus of attention OR external focus OR internal focus OR motor learning OR procedural learning OR teaching method OR learning method	performance OR learning OR proficien* OR mastery OR competenc* OR skills OR skill OR procedur* OR assessment OR comparative OR compare OR comparison OR measure* OR evaluat* OR educational measurement

* indicates a truncation search

### Measures of treatment effect and analysis

For all continuous outcomes means and standard deviations for all groups and all measures were extracted (this included baseline measures, post-acquisition -tests, retention tests and transfer tests). For continuous outcomes a pooled estimate of the standardized mean difference (SMD) with corresponding 95 % confidence intervals was estimated. Effect sizes were interpreted as described by Cohen (i.e. 0.2 represents a small effect, 0.5 a moderate effect and 0.8 a large effect) [21]. Statistical heterogeneity was evaluated with the I^2^ statistic [22]. With the help of I^2^ statistic it is possible to classify the proportion of effect estimates that can be attributed to heterogeneity between studies rather than sampling error [23]. I^2^ was classified accordingly to the guidelines presented in the Cochrane handbook for systematic reviews of interventions [24] (i.e.: 0 to 40 %: might not be important, 30 to 60 %: may represent moderate heterogeneity, 50 to 90 %: may represent substantial heterogeneity, 75 to 100 %: considerable heterogeneity).

### Assessment of risk of bias in included studies

Two reviewers independently evaluated the risk of bias of the included studies with the Cochrane Collaboration’s risk of bias tool [25]. After extraction of necessary data several sources of bias were evaluated (i.e. random sequence generation, allocation concealment, blinding of participants and personnel, blinding of outcome assessor, incomplete outcome data and selective reporting). The categories blinding of outcome assessor and incomplete outcome data were separately evaluated for the outcomes movement duration and procedure performance. Studies were classified as having a high risk of bias when at least one item was rated as high risk. An unclear risk of bias was assigned when at least one item was classified as unclear risk. And a low risk of bias was assigned when all items were rated as having a low risk.

## Results

### Results of the search

The search on electronic databases identified 874 potential records. It was possible to remove 134 duplicates. After screening of 740 titles and abstracts 686 records were excluded. The majority of records were excluded because of their intervention, a further 12 records were excluded due to study design and finally 4 records were excluded due to their population.

**Fig. 1:**
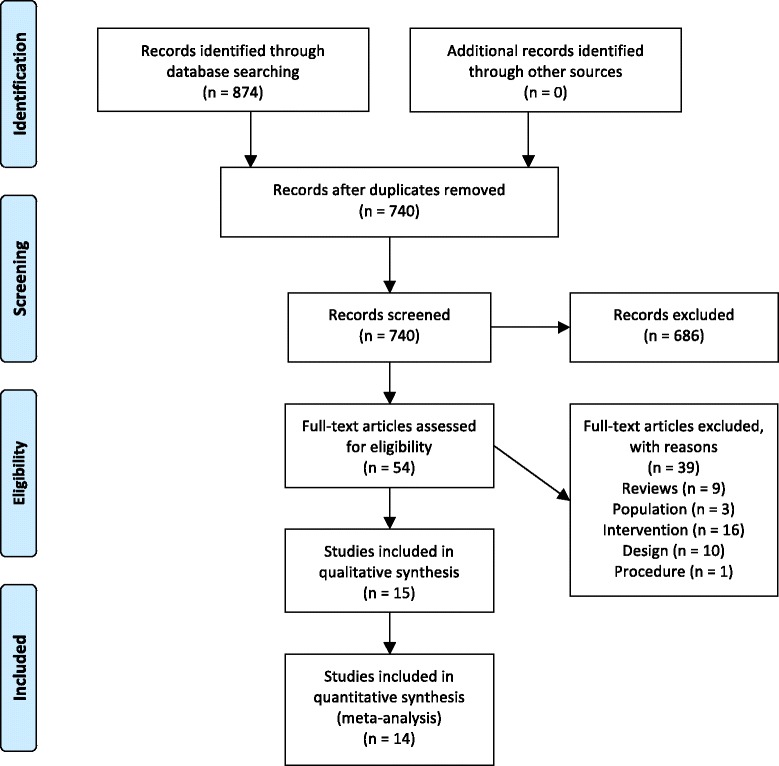
Study flow

The remaining 54 full-text articles were evaluated and 39 were excluded due to various reasons: Nine studies were reviews of primary studies [2, 13, 26–32]. Three studies recruited or described participants not matching the inclusion criteria [33–35]. Sixteen studies compared interventions not relevant for this review [36–51]. Ten studies used a design that was not eligible for this review [52–60]. One study trained a procedure that was not eligible for this review [61]. The remaining 15 studies were included for analysis in this review. An overview of the study flow during the selection process is presented in Fig. 1.

### Included studies

It was possible to include 15 studies with a total of 695 participants. All included studies were randomised controlled trials. In three studies part practice was compared against whole practice [9, 62, 63]. All three studies were performed in medical education. Because Brydges et al. [9] and Dubrowski et al. [62] included 3 arms (1^st^arm whole practice, 2^nd^ arm part practice (blocked), 3^rd^ arm part practice (random)) in their studies, they could also compare random practice against blocked practice. The influence of mental practice on procedural learning was evaluated by eight studies in the field of medical education [64–71]. Lastly, 4 studies evaluated whether terminal feedback or concurrent feedback was more beneficial for learning a procedure [72–75]. The first study [72] analysed the learning in undergraduate physiotherapy students. The last three studies were performed in medical education. A summary of the key characteristics of the 15 included studies is presented in Table 2.

**Table 2:** Characteristics of included studies

Motor learning principle	Study	Design/Setting	Population	Experience	Procedure	Educational intervention	Outcome measurement	Endpoints	Main findings
Part task practice-Whole task practice	Brydges et al. [9]	RCT (3 arms)/Canada	*N* = 18 post-graduate surgical residents (1st year)	Unclear experience	Orthopaedic surgical task (bone-plating task on artificial radial bones, with five separate skills)	Group 1: Whole task practice	Motion analysis system:	Pre-test	Post-acquisition-test:
						Group 2: Part task practice (random)	a) Number of hand movements	Post-acquisition test (5 min after training)	PT: Similar performance between groups
						Group 3: Part task practice (blocked)	b) Total time on task Videotape (expert evaluation):	Transfer test (1 week after the acquisition phase on an artificial radius)	MD: In favour of part practice (not significant)
							a) Global rating scale (operative performance)		Transfer test:
							b) 15-item checklist (operation-specific procedures)		PT: Infavour of part practice (not significant)
							c) Final product analysis		MD: In favour of part practice (not significant)
	Dubrowski et al. [62]	RCT (3 arms)/Canada	*N* = 28 medical students (1st and 2nd year),	Novice learners	Orthopaedic surgical task (bone plating task on artificial ulna bones, with five separate skills)	Group 1: Whole task practice (“functional- order-practice”) (3× 20 min sessions)	a) Checklist (operation-specific measurements)	Pre-test	Post-acquisition-test:
							b) Final product analysis	Post-acquisition test (immediately after acquisition phase)	PT: In favour of whole practice (significant)
						Group 2: Part practice (random) (3× 20 min. sessions)	c) Global rating scale (general operative performance)		
						Group 3: Part practice (blocked) (5× 12 min. sessions)	d) Duration of the drilling skill	Retention test (after 30 min rest period)	
						All participants practiced each skill 3 times for 2 min and 10 sec			
	Willaert et al. [63]	RCT/UK	*N* = 20 junior medical residents (surgery, radiology and cardiology)	Prior experience as an operator but not with this procedure	Carotid stenting procedure (virtual reality simulation)	Group 1: Part task rehearsal; 30 min of repeated catheterisations	a) Simulator derived dexterity metrics (procedure time, fluoroscopy time, contrast volume and number of roadmaps)	Post-acquisition test immediately after the training on a “real” patient	Post-acquisition-test:
						Group 2: Whole task rehearsal (*N* = 10); one full task rehearsal (~30 min)	b) Video recordings of hand movements (evaluated with a GRS and a PSRS)		PT: Similar performance between groups
							c) Non Technical Skills for Surgeons Rating Scale		MD: Similar performance between groups
							d) Self-assessment		
Random practice-Blocked practice	Brydges et al. [9]	See above	See above	See above	See above	See above	See above	Pre-test	Post-acquisition-test:
								Post-acquisition test (5 min after training)	PT: In favour of random practice (not significant)
								Transfer test (1 week after the acquisition phase on an artificial radius)	MD: In favour of random practice (not significant)
									Transfer test:
									PT: In favour of blocked practice (not significant)
									MD: In favour of random practice (not significant)
	Dubrowski et al. [62]	See above	See above	See above	See above	See above	See above	Pre-test	Post-acquisition-test:
								Post-acquisition test (immediately after acquisition phase)	PT: In favour of random practice (not significant)
								Retention test (after 30 min rest period)	
Mental practice	Arora et al. [64]	RCT/UK	*N* = 18 (surgeons)	Novices to laparoscopic surgery	Laparoscopic chole-cystectomies (simulation)	Group 1: Had an additional mental practice session before the simulation (30 min)	a) GRS of technical skills	Pre-test	Post-acquisition-test:
						Group 2: Had no additional training	b) Mental Imagery Questionnaire	Post-acquisition test	PT: In favour of mental practice (significant)
								Learning curve (all 5 practice sessions were measured)	
	Bathalon et al. [65]	RCT (3 arms)/Canada	*N* = 44 medical students (1st year)	Novices	Cricothyrotomy (simulation)	Group 1: Kinesiology practice (cognitive task analysis). The procedure was divided in 8 specific steps. All steps were discussed and practiced separately	OSCE examination:	Retention test (2 weeks after the teaching event)	Retention test:
							a) Knowledge of needed steps		MD: In favour of no mental practice (not significant)
						Group 2: Kinesiology and mental imagery. Same practice as group 1. With additional 5 min of mental imagery	b) Time and fluidity of intervention		
						Group 3: Standard educational ATLS approach			
	Geoffrion et al. [66]	Multi-centre RCT/8 centres across Canada and the USA	*N* = 50 junior gynaecology residents	All participants were at the start of their learning curve	Vaginal hysterectomies	Group 1: Mental Practice. The MP script enumerated the procedure steps based on a reference textbook. The participants performed the MP with an expert educator. MP was continued individually until the participant felt comfortable with the procedure. Group 2: Participants were encouraged to read a textbook describing the procedure.	a) GRS of surgical skill	Pre-test	Post-acquisition-test:
							b) Procedure-specific score	Post-acquisition test (immediately after the intervention)	PT: In favour of mental practice (non significant)
							c) Self-assessment (GRS)		
							d) Self-confidence		MD: In favour of mental practice (not significant)
							e) Time in operating theatre		
							f) Attending surgeons evaluations (e.g. blood loss and complications)		
	Jungmann et al. [67]	RCT/Germany	*N* = 40 medical students	Novice learners	Laparoscopic exercises:	All participants followed 2 sessions on a simulator with three tasks.	Performance measures:	Pre-test (parameters of the 1st training session)	Post-acquisition-test:
					a) Grasping movements	Between the 2 sessions:	a) Time		
					b) Tissue manipulation	Group 1: Additional mental practice (at least 4 times and not less than 3 min)	b) Tip trajectory		
					c) Surgeons’ Knot	Group 2: No additional training	c) Time of the instrument collision	Post-acquisition test (parameters of the 2nd training session)	MD: In favour of no mental practice (not significant)
							Visual-spatial ability:		
							a) Cube test		
	Komesu et al. [68]	Multi-centre RCT/6 academic centres in the USA	*N* = 68 gynaecology residents	Some prior experience with the procedure	Cystoscopy	Group 1: Mental practice 24-48 h prior to a scheduled cystoscopy. Session lasted < 20 min	a) Global Scale of Operative Performance	1st Post-acquisition test (Evaluation of the 1st procedure)	Post-acquisition-test:
							b) Time required for cystoscopy		PT: In favour for mental practice (significant)
						Group 2: Students were encouraged to read a standard text 24-48 h prior to a scheduled cystoscopy.	c) Competence to perform the procedure		
							d) Preparedness for the procedure	2nd Post-acquisition test (Evaluation of the 2nd procedure)	MD: In favour of no mental practice (not significant)
	Rakestraw et al. [69]	RCT/USA	*N* = 160 medical students (2nd year)	Novice learners	Pelvic examination	Group 1 (control group): 1 student practiced the task and two students observed the performance	Knowledge of attainment	1st post-acquisition test (after practice on models)	Study not included into the meta-analysis
							a) Memory list of relevant steps		
						Group 2: Mental practice before the task (pre-motor).	b) Patient record		
						Group 3: Mental practice after the task (post-motor)	Performance measures:	Retention test (immediately before the evaluation on a simulated patients)	
							a) Behavioural checklist		
						Group 4: Mental practice before and after the task.			
								Transfer test (simulated -patients)	
	Sanders et al. [70]	RCT (3arms)/USA	*N* = 65 medical students (2nd year)	Unclear experience	Cutting and suturing a pig’s foots	Group 1: 3 sessions of physical practice	a) 7-item GRS	Post-acquisition-test (During the 1st training session)	Post-acquisition-test:
						Group 2: 2 sessions of physical practice and 1 session of mental practice (relaxation exercises and imagery exercises)	b) Surgical skills attitude questionnaire (Confidence)		PT: In favour of mental practice (not significant)
								Transfer test (10 days after the last session)	Transfer test:
						Group 3: 1 session of physical practice and 2 sessions of mental practice (relaxation exercises and imagery Tr test: exercises)			PT: In favour of no mental practice (not significant)
	Sanders et al. [71]	RCT/USA	*N* = 64 medical students (2nd year)	Unclear experience	Cutting and suturing a pig’s foot	Group 1: Mental practice for ~30 min (1st part relaxation exercises and 2nd part imagery exercises) (2 sessions)	Surgical performance:	Pre-test (confounding)	Post-acquisition-test:
							a) 15 item checklist (surgical behaviour)		
							b) 6 specific rating scales		
						Group 2: Textbook study for 30 min (using a verbal method) (2 sessions)	Measurement of confounding:	Post-acquisition test (after the 1st intervention period)	PT: In favour of no mental practice (not significant)
						Afterwards: All participants received 1 h practice under supervision (together)	a) Self-confidence	1st retention test (after the 1 h practice session)	Transfer test:
							b) Prior learning		PT: In favour of mental practice (not significant)
							c) Anxiety	2nd retention test (10 days after the last intervention)	
							d) Visual-spatial ability		
Terminal Feedback-Concurrent Feedback	Chang et al. [72]	RCT (3arms)/Taiwan	*N* = 36 undergraduate physical therapist students	Limited exposure to peripheral joint mobilisa-tion	Joint mobilization (simulation)	Group 1: Received concurrent graphical feedback on their performance during three 25 trials blocks	Accuracy of performance:	Pre-test	Post-acquisition-test:
							a) Deviation of the grading force	Acquisition phase test	PT: In favour of terminal feedback (not significant)
						Group 2: Received terminal feedback on their performance after each trial block		Post-acquisition test (10 min after the acquisition phase)	
									Retention test:
						Group 3: Received no feedback		Retention test (5 days after the acquisition phase)	PT: In favour of concurrent feedback (not significant)
						The skill acquisition phase lasted ~40 min for all groups			
	Gofton et al. [73]	RCT (3 arms)/Canada	*N* = 45 surgical residents (1st or 2nd year) or senior medical students	Some prior experience with the procedure	Acetabular cup placement (simulation)	Group 1: Conventional training	Performance measures:	Pre-test	Post-acquisition-test:
						Group 2: Received concurrent feedback during each trial	a) Acetabular position	Post-acquisition test & transfer test	PT: In favour of terminal feedback (not significant)
							b) Time required to determine optimal position		
						Group 3: Received terminal feedback after every trial	Visual-spatial ability	(10 min after the skill acquisition)	Retention test:
							a) Mental Rotations Test Part A	Retention- & transfer test (6 weeks after the skill acquisition)	PT: In favour of concurrent feedback (not significant)
	O’Connor et al. [74]	RCT (3 arms)/USA	*N* = 9 medical students (1st and 2nd year)	Unclear experience	Laparoscopic knot-tying and suturing (simulation)	Group 1: Received no feedback during the 4 weeks	Measurement of performance:	Measurement points during all practice sessions	Post-acquisition-test:
							a) Time		PT: In favour of concurrent feedback (not significant)
						Group 2: Received KR at the end of each practice session	b) Instrument path length		
							c) Smoothness of instruments		
						Group 3: Received KR and KP during and at the end of each practice session	d) Examination of each knot		
							f) Error scale		
	Walsh et al. [75]	RCT/Canada	*N* = 30 medical students (1st and 2nd year)	Novice learners	Colonoscopy (simulation)	Group 1: Received concurrent feedback (KP)	Performance measures:	Pre-test	Post-acquisition-test:
							a) Execution time	Post-acquisition test (immediately after the practice)	PT: In favour of concurrent feedback (not significant)
						Group 2: Received terminal feedback (KR)	b) 5-item Checklist (endoscopic performance)		
									MD: In favour of concurrent feedback (not significant)
							c) GRS	2nd retention test (1 week after the intervention)	
									Retention test:
								Transfer test (1 week after the intervention)	PT: In favour of concurrent feedback (not significant)
									MD: In favour of concurrent feedback (not significant)
									Transfer test:
									PT: In favour of terminal feedback (significant)
									MD: In favour of terminal feedback (significant)

*ATLS* Advanced Trauma Life Support, *GRS* Global Rating Scale, *KP* Knowledge of performance, *KR* Knowledge of results, *MD* Movement duration, *MI* Mental imagery, *mMIQ* modified Mental Imagery Questionnaire, *MP* Mental practice, *PSRS* Procedure Specific Rating Scale, *PT* Performance tests, *VH* Vaginal hysterectomy

### Findings

#### Whole practice - part practice (WP-PP)

After the search three studies were included for the comparison whole practice against part practice. The procedure that was trained was either an orthopaedic surgical skill [9, 62] or a carotid stenting procedure [63] (see Table 2 for details).

### Performance tests WP-PP

**Fig. 2:**
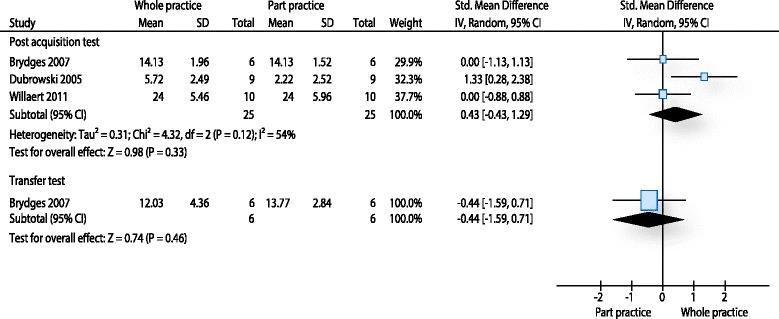
Analysis performance tests whole practice - part practice. The forest plot relates to the outcome performance test. The outcome movement duration is not illustrated

Three studies [9, 62, 63] provided data for this outcome. All studies used procedure specific checklists to measure the effect of the intervention on orthopaedic surgical tasks [9, 62] or on a carotid stenting procedure [63]. The results of a post-acquisition test (with 50 participants) immediately after the intervention showed a moderate effect size of 0.43 SMD (95 % CI -0.43 to 1.29) in favour for whole practice (p: 0.33). However, heterogeneity was considerable for this analysis (I^2^: 54 %) (Fig. 2). Only Brydges et al. [9] measured the procedure on a transfer test (a cadaver bone was used instead of an artificial bone). The results of their study were in favour for part practice (SMD: -0.44, 95 % CI -1.59 to 0.71, p: 0.46).

### Movement duration WP-PP

Two studies [9, 63] measured the time needed to perform the procedure. Both studies measured this outcome with a post-acquisition test within 5 min after the intervention for learning the procedure ceased. In total 32 participants were included for the post-acquisition test. The pooled effect size was 0.03 SMD (95 % CI -0.67 to 0.72, p: 0.93). Heterogeneity measured with I^2^ was low (0 %). One study [9] measured results on a transfer test. The effect size of the transfer test was in favour for part practice (SMD: 0.30, 95 % CI -0.84 to 1.44, p: 0.61).

### Random practice - blocked practice (RP-BP)

#### Performance tests RP-BP

**Fig. 3:**
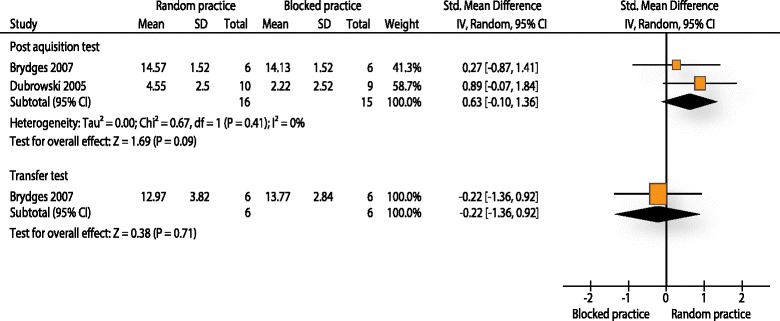
Analysis performance tests random practice - blocked practice. The forest plot relates to the outcome performance test. The outcome movement duration is not illustrated

Brydges and colleagues [9] and Dubrowski et al. [62] used procedure specific checklists to measure skill performance of orthopaedic surgical procedures (i.e. bone-plating task). Both studies measured performance on a post-acquisition test within 5 min shortly after the practice session. For the post-acquisition test 31 participants were included. The effect size was moderate (SMD: 0.63) and in favour for random practice (95 % CI -0.10 to 1.36). However, the result was statistically not significant (p: 0.09). Heterogeneity between studies was low (I^2^: 0 %). Brydges et al. also measured the procedure on a transfer test. The results of the transfer test were in favour for the blocked practice but were statistically not significant (SMD: -0.22, 95 % CI -1.36 to 0.92, p: 0.71). Because only Brydges et al. was included for this analysis, a pooling was not possible (Fig. 3).

### Movement duration RP-BP

One study [9] evaluated the effectiveness of a random practice intervention against a blocked practice intervention on the outcome movement duration for an orthopaedic surgical procedure (bone-plating). Twelve participants were analysed for this outcome. Effect sizes were small and close to zero (SMD: -0.16, 95 % CI -1.29 to 0.98 for a post-acquisition test and SMD: -0.06, 95 % CI: -1.20 to 1.07 for a transfer test).

### Mental practice (MP)

After the selection process eight studies were included for this comparison. Five studies compared mental practice against a standard educational intervention (e.g. textbook readings) [65, 66, 68, 69, 71]. Two studies compared the effect of additional mental practice against no additional practice [64, 67]. One study [70] compared different quantities of mental practice and physical practice (see Table 2 for greater detail of interventions). All procedures with one exception were related to surgical education. The procedure outside the surgical domain was pelvic examination [69]. Two studies evaluated the influence of mental practice on basic surgical skills [70, 71]. Two studies trained laparoscopic procedures [64, 67]. Two studies evaluated the influence of mental practice in relation to surgical procedures in gynaecology [66, 68] and Bathalon and colleagues [65] were interested whether mental practice could have a beneficial influence on learning of a cricothyrotomy procedure.

### Performance tests (MP)

**Fig. 4:**
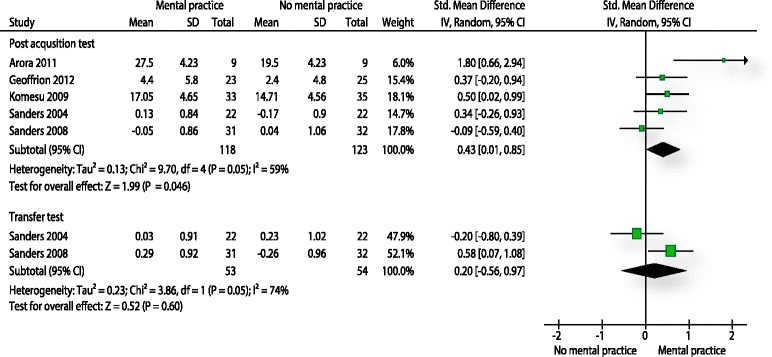
Analysis performance tests mental practice. The forest plot relates to the outcome performance test. The outcome movement duration is not illustrated

Five studies [64, 66, 68, 70, 71] evaluated procedural skills with a performance test. In four studies the outcome measure was a global rating scale. Sanders et al. [71] used a combination of several specific rating scales. In total 241 participants were analysed. The pooled effect size was small to moderate (SMD: 0.43, 95 % CI 0.01 to 0.85) in favour of mental practice on a post-acquisition test. Furthermore, the result was statistically significant (p: 0.046). Heterogeneity was moderate (I^2^: 59 %). Two of the above mentioned studies measured procedural performance also on a transfer test [70, 71]. Both studies provided data from 107 participants. The pooled estimate of the effect was small (SMD: 0.20, 95 % CI -0.56 to 0.97) and in favour for the mental practice group (Fig. 4). Furthermore, the effect was statistically not significant (p: 0.60) and heterogeneity was considerable (I^2^: 74 %).

### Movement duration (MP)

It was possible to include four studies for the outcome movement duration [65–68]. All measured the effect of mental practice on a post-acquisition test. The post-acquisition test was measured shortly or immediately after the intervention period. Only Bathalon and colleagues [65] scheduled their test two weeks after the intervention. In total 181 participants were analysed for this outcome. The pooled estimate was 0.00 SMD with a 95 % CI between -0.29 and 0.30. The result was statistically not significant (p: 0.98) and heterogeneity was low (I^2^: 0 %).

### Augmented feedback (AF)

Four studies compared different ways of giving feedback [72–75]. One study was based in a physiotherapeutic setting and evaluated whether learning of a joint mobilisation procedure benefitted more from terminal or a concurrent feedback. Gofton et al. [73] trained an orthopaedic surgical procedure with surgical residents and feedback was given as concurrent or terminal feedback. Walsh et al. [75] evaluated the learning of a colonoscopy procedure in medical students after receiving concurrent or terminal feedback. The study of O’Conor and colleagues [74] trained a laparoscopic procedure in medical students.

### Performance tests (AF)

**Fig. 5:**
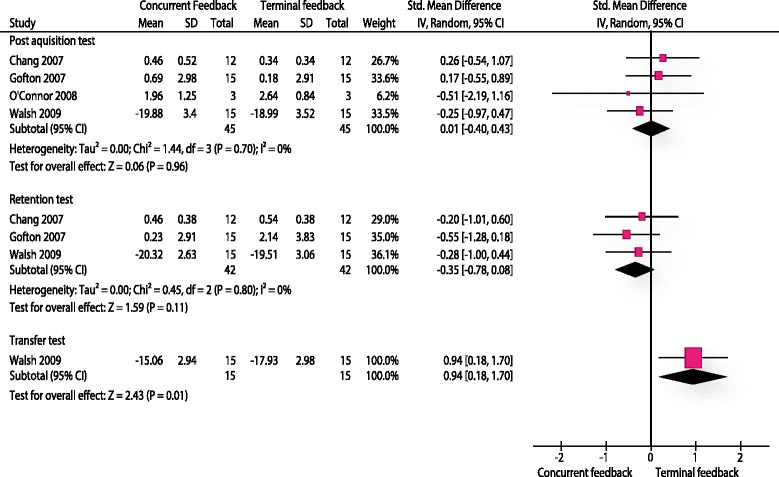
Analysis performance tests terminal feedback - concurrent feedback. The forest plot relates to the outcome performance test. The outcome movement duration is not illustrated

All four studies evaluated procedural skills. One study used a procedure specific checklist [75]. The remaining three studies measured this outcome with error scores. It was possible to compare three different endpoints. A first post-acquisition test shortly after the intervention (0-10 min after the last session) was measured by all four studies. In total 90 participants were included for this analysis. The pooled effect size for this analysis was 0.01 SMD (95 % CI: -0.46 - 0.33) and statistically not significant (p: 0.75). In addition three studies also measured a delayed retention test [72, 73, 75]. Results were homogenous (I^2^: 0 %). The pooled estimate for this analysis was -0.35 SMD (95 % CI: -0.78 - 0.08) in favour for the concurrent feedback group and statistically not significant (p: 0.11). One study [75] with 30 participants measured procedural skills on a transfer test. They presented a large effect size in favour for the terminal feedback group (SMD: 0.94, 95 % CI 0.18 to 1.70) (Fig. 5).

### Movement duration (AF)

Walsh et al. [75] presented data for this outcome. They evaluated three endpoints. An immediate post-acquisition test was in favour of the concurrent feedback group -0.48 SMD (95 % CI -1.21 to 0.25, p: 0.19). A delayed retention test (1 week after the intervention) was in favour for the concurrent feedback group as well (SMD: -0.20, 95 % CI -0.91 to 0.52, p: 0.59). Lastly, the results of a transfer test were clearly in favour for the terminal feedback group (SMD: 0.74, 95 % CI 0.00 to 1.48, p: 0.047).

### Risk of bias assessment

**Fig. 6:**
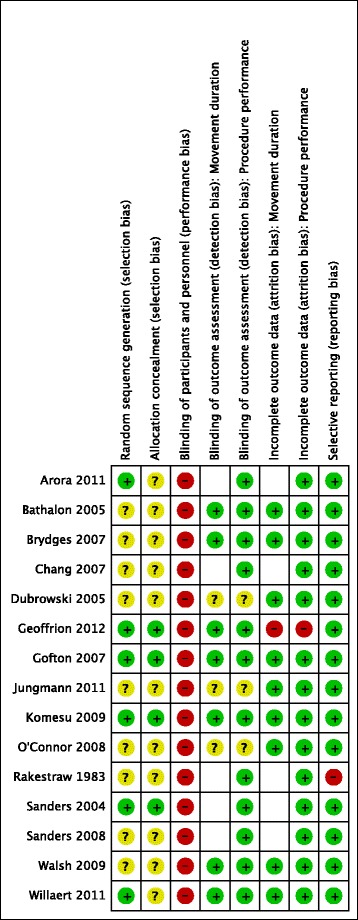
Risk of bias evaluation

All 15 included studies were evaluated on their risk of bias (Fig. 6). All studies had a high risk of bias because they didn’t blind leaners and educators. Therefore, a performance bias must be assumed in all studies. All studies reported that they randomly generated groups but the method of the random sequence generation was often poorly reported. Furthermore, only four studies [66, 68, 70, 73] were judged with a low risk on allocation concealment. Therefore, a selection bias might have occurred in the majority of studies. A detection bias might have occurred in three studies [62, 67, 74] they were appraised as having an unclear risk of bias with regard to the blinding of outcome assessors. Five studies [64, 69–72] did not measure the outcome movement duration. Therefore, the corresponding items were not evaluated.

## Discussion

### Summary of main results

This review set out to explore the question, if teaching of procedural skills based on motor learning principles is effective for skill acquisition and skill retention in physiotherapy and medical education? Four different motor learning principles were evaluated. We could include 15 studies in this systematic review. The majority of studies investigated use of mental practice (*n* = 8). Only two studies compared random practice against blocked practice, three studies evaluated part practice against whole practice and four studies investigated augmented feedback.

The comparison whole practice versus part practice showed no statistically significant results. Despite being not significant, performance tests indicated that results of a post-acquisition test were in favour for whole practice, therefore possibly indicating that WP improves immediate performance of skill after a period of training. For longer term outcome, performance seemed to be more effective on a transfer test when a part practice regime was followed. Effect sizes were small to moderate on the transfer test. Only one study [9] used a transfer test to evaluate the effectiveness of the intervention on a similar procedure. Three studies and therefore considerably more participants were available for the post-acquisition test. However, post-acquisition tests provide only limited evidence of learning and the observed changes may be related to transient changes in performance and it is difficult to estimate the amount of learning that has occurred with these tests. Little educational diversity was present with regard to the length of the time interval between the intervention and the administration of the post-acquisition test (i.e. measured immediately or 5 min after the intervention). In contrast educational diversity was present with regard to the participant’s level of experience. Experience ranged between novices [62] and some experience in a related procedure [63]. This is of particular importance because part practice might be helpful for novice learners [76]. According to motor learning theory a part practice approach might be applicable for skill learning due to a reduced intrinsic load of the task for the learner. Especially novice learners might benefit from a load reducing approach, which increases the resources available for the learning process itself [76]. In contrast learners with a higher skill level are assumed to benefit less from a part practice schedule [77].

The evaluation of random practice against blocked practice did not show a statistically significant result. Random practice appeared more beneficial for immediate performance after a period of training, however this improvement did not persist on a transfer test. This effect in the opposite direction of the expected direction might be explained by the complexity of the procedures. Effects of random versus blocked practice are a relatively robust phenomenon in simple tasks [78]. However, evidence is less clear with regard to complex tasks [78]. Both included studies trained procedures that can be classified as complex, which may have caused the unexpected result. However, task complexity differed between the test conditions. An artificial bone was used during the acquisition phase and for the post-acquisition tests. Brydges et al. [9] reported that the complexity of the task was moderate with regard to the skill level of the participants. Random practice might have positively influenced immediate performance because task complexity for the learners was only moderate and learners might have benefitted from deeper and more elaborative memory processes (i.e. a more intense motor planning) caused by random practice. During the transfer test a cadaver bone was used and complexity was significantly increased for the participants. The higher task complexity of the transfer test compared to the complexity of the acquisition phase might have prevented the participants to fully benefit from random practice. A similar finding of a reversed effect of random versus blocked practice on transfer tests was reported by Albaret and Thon [79] when they considerably increased the complexity of practiced tasks. With regard to educational diversity both studies were relatively homogenous. This included the use of similar measurement scales and procedures.

The use of mental practice resulted in significant increases of performance on a post-aquisition test. A transfer test was in favour for mental practice but did not reach the level of significance. No statistically significant results were found for the outcome movement duration. The effectiveness of mental practice on performance tests included five randomised controlled trials. Educational and methodological heterogeneity was considerable for this comparison. Most importantly in two studies [64, 67] there was no active comparator. This might have introduced a bias in favour of mental practice. This is especially true for the study of Arora and colleagues [64]. There was diversity in relation to the included participants. The spectrum ranged from undergraduate medical students to surgical and gynaecology residents. Furthermore, the participants experience varied between no prior experience to some experience with the procedure. Little heterogeneity was present for the measurement and all studies measured the post-acquisition test immediately after the training period.

Performance was statistically significant better when the feedback was given as terminal feedback on a transfer test. Concurrent feedback seemed to be superior on a delayed retention test with regard to the outcome performance tests. However, the finding did not reach the level of statistical significance. The superiority of the terminal feedback on the transfer test might be explained by the guidance hypothesis [80], which states that initial performance can benefit from frequent feedback but in later stages learners might develop a dependency on feedback and therefore performance on a transfer test without feedback might be reduced. However, the guidance hypothesis cannot explain the findings of the delayed retention test. Performance of procedures was measured differently compared with the other three comparisons. Three studies used participant’s errors [72–74]. Only one study [75] used a procedure specific checklist. The procedure that was trained differed because one study [72] was based in physiotherapy and the remaining three procedures were surgical procedures. The participants were either students or surgical residents. Their experience level ranged between novice learners to some prior experience. Furthermore, there was considerable diversity with regard to the length of the retention interval of the delayed retention test. The time point of measurement ranged between five days [72] and 6 weeks [73].

### Quality of the evidence

The risk of bias of included studies was universally high. This was inevitable because a blinding of learners and educators was difficult or nearly impossible achieve for these interventions. Furthermore, all included studies claimed to be randomised controlled studies. But only four studies [66, 68, 70, 73] sufficiently described the process of randomisation. The chance of selection bias is significantly reduced with a randomised controlled trial design. But when the selection procedure is not described in detail it is unclear whether this important threat to internal validity is avoided. It was not possible to exclude a detection bias in this review, because blinding of outcome assessors wasn’t explicitly reported by all studies. As blinding of outcome assessors is especially important for subjective outcome measures the outcome procedure performance is probably at higher risk to systematic measurement error than the outcome movement duration.

### Potential biases in the review process

The strength of this review was the systematic procedure. Studies were selected with clearly defined inclusion and exclusion criteria. Risk of bias of all studies was assessed using the Cochrane’s risk of bias tool [25] and it was possible to perform a meta-analysis for all comparisons and for all outcome measures. One weakness of this review was that it was necessary to extract data from several studies from graphical representations as numerical data were not available [9, 72, 73, 75]. It is not possible to exclude any imprecision from this process. However, arguably any imprecision might have occurred in both directions.

A further limitation of this review was that only few studies and participants could be included in the analysis. Especially, the comparisons WP - PP (three studies) and RP - BP (two studies) might suffer from a small study bias [81]. Furthermore, the following features might have influenced the findings.

The majority of studies used a simulated environment and only MP was also applied in real world practice [66, 68]. Educational dimensions may differ between simulation and practice. Application of the procedures in real practice may also involve other dimensions than solely procedural skills (e.g. dimensions such as communication and decision-making). Therefore, learners and educators might vary their strategies to train a procedural skill depending on whether other dimensions were also included in the training. Furthermore, assessment methods varied between simulation based training (e.g. computed based metrics [67]) and practice based training (e.g. attending surgeons evaluation [66]). This might have introduced a bias in the MP findings. A limitation of the other three comparisons is that the transfer of the evidence into practice needs to be further evaluated.

A further limitation of this review is that the spectrum, of included learners ranged between undergraduates (novices) and postgraduates (experts). All studies aimed to train a novel procedure. However, learning might be different in novices and experts. Latter might benefit from transfer of learning from previous learned similar procedures. This limitation might especially concern the findings of the WP-PP analysis.

Lastly, task complexity varied between procedures. All of the procedures can be classified as reasonable complex procedures because they fulfil at least two features of complex procedures when the framework of Wulf and Shea [78] is used. Firstly, it is not likely to learn them in a single session. Secondly, all procedures involve movements of more than one degree of freedom. But the last feature of complex procedures (i.e. ecological validity) was not completely fulfilled by the simulation studies, because they are trained in an artificial environment. This may affect the analysis of MP, because highly complex real world procedures were analysed together with complex simulation procedures.

### Agreements with other studies

The finding from this review, that part practice was not superior to whole practice on a retention test is also supported by a meta-analysis of Wickens et al. [77]. Their review was related to the field of military procedures and therefore findings are only partial comparable to this review. The authors reported that part practice had limitations in some of their included studies. Especially, when parts of a procedure were created by fractionation they observed a failure of part practice. This might have lead to a separation of time dependent parts and learners possibly did not develop relevant time-sequencing skills [77].

The finding of this review that mental practice is effective is supported by studies in related fields. Already in 1988 Feltz and Landers showed that motor imagery has a positive effect on skill learning [82]. More recently Braun et al. [83] showed that mental practice also had some beneficial influence on skill learning in a population with stroke survivors. A concept why mental practice may be effective for the learning of procedures was introduced by Jeannerod [84] with the functional equivalence hypothesis. This theory is build upon the assumption that when a movement is imagined, the brain activity is similar to the brain activity of a physical execution of this movement. Hétu et al. [85] supported the theory in a meta-analysis by identifying a large neural network in motor related regions that is activated by mental practice. However, the primary motor cortex, which is normally active during physical practice, was not consistently activated during mental practice. This indicates that mental practice can be seen as a support of physical practice and not a replacement.

A recent systematic review [29] evaluated the role of augmented feedback for procedural learning in medical education. Their findings were similar to this review. However, they didn’t analyse a transfer test, which was in favour of terminal feedback.

Finally, while all the studies included in this review related to the teaching and acquisition of complex motor skills, only one of the 15 studies specifically referred to physiotherapeutic procedures. Therefore, any inferences in relation to structuring of teaching and practice of complex therapeutic motor skills should be made with extreme caution.

## Conclusions

There is some evidence to recommend the use of mental practice for procedural learning in medical education. Especially, surgical skills benefitted from mental practice. In order to improve learning of procedures this motor learning principle should be considered for implementation. There is limited evidence to conclude that terminal feedback is more effective than concurrent feedback on a transfer test. However, only one study showed this effect and future studies need to support this finding. Therefore, it may be justified to cautiously use this kind of feedback. There were indications that whole training has some advantages over part training on immediate post-acquisition tests. However, evidence was not strong enough to justify the integration of this principle in curricula. The same relates to the use of random practice. The limited evidence of improved performance on post-acquisition tests might support the use of this principle in some circumstances. In addition, educators should be aware that it is not safe to make inferences about learning with post-acquisition tests. This should encourage faculty to implement delayed retention and transfer tests to assess the learning of procedures.

The evidence available for the reviewed motor learning principles is not strong enough to draw strong conclusions about effectiveness, therefore there is a need for more studies with adequate design (i.e. randomised controlled trials) and sufficient sample size. With the exception of the principle mental practice, less than five randomised controlled studies were available for analysis for each of the selected motor learning principles. Furthermore, sample sizes of the studies were small and only two studies (both for the principle mental practice) had sample sizes over 30 participants per trial arm. Most studies evaluated the application of motor learning principles in surgical education. Therefore, there is a demand for research in other HPE settings where complex procedural skills are taught.

## Additional file

Additional file 1:
**Practical application of the motor learning principles.** (DOCX 110 kb)
