# Supplementary material for: A systematic review and meta-analysis of selected motor learning principles in physiotherapy and medical education
Source: BMC Med Educ. 2016 Jan 15;16:15. doi: 10.1186/s12909-016-0538-z (PMC4714441; doi:10.1186/s12909-016-0538-z)
Supplement: Additional file 1: — Practical application of the motor learning principles. (DOCX 110 kb) [file 12909_2016_538_MOESM1_ESM.docx]

## Practical application of the motor learning principles

To illustrate the motor learning principles an example from the education in neurological physiotherapy is used.

| The procedure relates to the learning of a sit to stand movement. Within this portfolio the procedure and relevant therapeutic aspects are used from an Australian guideline [1]. The authors evaluated that the sit to stand movement can be divided into a pre-extension and an extension phase. The transition between the two phases occurs when the thighs leave the supporting surface. During the pre-extension phase participants should place their feet backward. Furthermore, a trunk rotation in a forward direction might be important during this phase. In the extension phase participants generate a vertical body movement by extending hip, knee and ankles in a coordinated movement synergy. During the teaching of that procedure physiotherapists can provide instructions or feedback with regard to movement characteristics. Physiotherapists might also intervene to ensure patient’s safety by providing stability to various body segments (e.g. the knee joint during the extension phase). Depending on the patient’s presentation physiotherapists might use various interventions to improve the sit to stand movement. | |
| --- | --- |
| **Part practice** | **Whole practice** |
| When a part practice approach is used, students would train the pre-extension phase separately from the extension phase. During the pre-extension phase analysis of the movement pattern, possible feedback and correction would be practiced. Students might be encouraged to train the pre-extension phase 5 times.  Afterwards, relevant therapeutic aspects would be trained in the extension phase. This part might be trained 5 times as well. | In contrast, when a whole practice schedule is applied the procedure would be trained without separation between the pre-extension and extension phase. |
| **Random practice** | **Blocked practice** |
| The procedure can be randomly practiced with similar procedures such a transfer from wheelchair to a bed or going down on a mat. During a 30 minute class every of the three procedures is practiced 10 times in a random order. | The blocked part practice would start with 10 repetitions of the sit to stand procedure and afterwards the remaining two procedures would be practiced. |
| **Mental practice** | **No mental practice** |
| Students receive a general teaching class about the sit to stand movement. The movement and its relevant parts (e.g. pre-extension phase and extension phase) are discussed. Afterwards, the performance of patients with various clinical signs on the sit to stand movement is illustrated. For example sit to stand movement characteristics of a patient with a paresis of the left knee muscles are explained. Further examples are discussed (e.g. paresis of trunk muscles or the plantar flexors). This introductory teaching class is finished with presentation of possible therapeutic interventions (e.g. providing external knee stability or movement corrections). After this teaching event a mental practice intervention with several steps is trained.  1) The procedure and its relevant parts are presented on pictures.  2) Students are encouraged to close their eyes and to imagine the sit to stand movement from an internal point of view (internal point of view for the participant).  3) Videos from patients with various clinical signs are presented (e.g. a patient with a paresis of the left quadriceps).  4) Students are encouraged to close their eyes and to imagine the movement from a therapeutic point of view (i.e. internal point of view for the therapist).  5) The video from the same patient is presented again but this time a therapist is present and uses a therapeutic intervention.  6) Students are encouraged to imagine the intervention from an internal therapeutic point of view.  7) Steps 3 to 6 are repeated with different patients. | - |
| **Terminal feedback** | **Concurrent feedback** |
| Students are asked to practice the procedure 5 times. The educator provides feedback after the 5^th^ practice trial. The feedback is a summary feedback about all five trials. For example the performance of the procedure is measured on a checklist (with important features). After the last trial the educator analyses the checklists and reports to the student the checklist items with the best and worst performance. | Students are asked to practice the same amount of repetitions. But augmented feedback can be provided during every trial. |

## References

1. Carr JH, Shepherd RB: **Stroke Rehabilitation: Guidelines for Exercise and Training to Optimize Motor Skill**. Edinburgh: Butterworth-Heinemann; 2003.
